# Better Sleep in a Strange Bed? Sleep Quality in South African Women with Posttraumatic Stress Disorder

**DOI:** 10.3389/fpsyg.2017.01555

**Published:** 2017-09-12

**Authors:** Gosia Lipinska, Kevin G. F. Thomas

**Affiliations:** UCT Sleep Sciences and Applied Cognitive Science and Experimental Neuroscience Team, Department of Psychology, University of Cape Town Cape Town, South Africa

**Keywords:** polysomnography, posttraumatic stress disorder, sleep, sleep-state misperception, subjective sleep quality

## Abstract

Although individuals diagnosed with posttraumatic stress disorder (PTSD) regularly report subjective sleep disruption, many studies using objective measures (e.g., polysomnography) report no PTSD-related sleep disruption. To account for these inconsistencies, some authors hypothesize that PTSD-diagnosed individuals have sleep-state misperception; that is, they self-report experiencing poor sleep quality, but objectively sleep relatively normally. We tested this sleep-state misperception hypothesis, collecting data on subjectively-reported sleep quality (in the home, and in the laboratory) and on objectively-measured, laboratory-based, sleep quality in PTSD-diagnosed participants from low socioeconomic status South African communities. Women with PTSD (*n* = 21), with trauma exposure but no PTSD (TE; *n* = 19), and healthy controls (HC; *n* = 20) completed questionnaires on their average sleep quality in the past 30 days, and on their sleep quality after a night (8 h) of polysomnographic-monitored sleep in the laboratory. PTSD-diagnosed individuals reported poorer everyday subjective sleep quality than TE and HC individuals. In the laboratory, however, there were no between-group differences in subjective sleep quality, and few between-group differences in objective sleep quality (PTSD-diagnosed individuals only had decreased sleep depth). Furthermore, whereas measures of laboratory-based objective and subjective sleep quality correlated significantly, especially in PTSD-diagnosed individuals, there were few significant associations between objective sleep measures and everyday subjective sleep quality. Taken together, these findings suggest that PTSD-diagnosed individuals likely experienced better sleep quality in the laboratory than at home. Descriptive observations corroborated this interpretation, with almost half the sample rating their laboratory sleep (which they described as “safe” and “quiet”) as better than their home sleep (which was experienced in an atmosphere marked by high levels of violence and nighttime noise). These findings disconfirm the sleep-state misperception hypothesis as related to PTSD, and suggest that the laboratory environment may influence sleep quality positively in these individuals. Many investigations of sleep in PTSD do not consider the influence of the laboratory environment. Our findings suggest that future studies in this field should consider that sleep-state misperception may be an artifact of the laboratory setting, especially when samples are drawn from communities where violence and crime are an everyday reality.

## Introduction

Sleep difficulties, clinically characterized by insomnia and nightmares and usually assessed via self-report, are key diagnostic criteria for PTSD (Koren et al., 2002; APA, 2013; Taylor et al., 2014; Gehrman et al., 2015). Even before the diagnostic category was formally constituted, however, many individuals who had experienced trauma (e.g., combat veterans) reported psychological distress as a consequence of frequent night-time wakefulness and night terrors. For instance, many soldiers who participated in the great European military of conflicts of the 17th and 18th centuries were described by military physicians as experiencing “nostalgia,” a condition that included sleep disruption and insomnia amongst other symptoms such as melancholy, loss of appetite, anxiety, and cardiac palpitations (Crocq and Crocq, 2000). Even centuries earlier than that, ancient texts described, in graphic terms, how traumatic events were re-experienced in dreams (Lucretius' De Rerum Natura, Book IV, transl. William Ellery Leonard; Crocq and Crocq, 2000):

The minds of mortals… often in sleep will do and dare the same… Kings take the towns by storm, succumb to capture, battle on the field, raise a wild cry as if their throats were cut even then and there. And many wrestle on and groan with pains, and fill all regions round with mighty cries and wild, as if then gnawed by fangs of panther or of lion fierce.

Since the contemporary conception of PTSD was introduced to the psychiatric nomenclature a little more than three decades ago, numerous empirical studies have documented the presence of subjective sleep difficulties in individuals who meet formal diagnostic criteria (Kinzie et al., 1984; Mellman et al., 1995a; Giosan et al., 2015; Werner et al., 2016). For instance, Giosan et al. (2015) interviewed more than 2,000 men ~29 months after their deployment to the World Trade Center in the days following the 9/11 attacks. They found that those diagnosed with PTSD as a consequence of their deployment experiences reported, relative to those without PTSD, more insomnia and nightmares, more instances of waking feeling unrefreshed, as well as more difficulties with sleep maintenance and snoring. These subjectively reported sleep difficulties were significantly associated with functional disability at work, in the home, and socially. Werner et al. (2016) found similar results using an entirely different cohort of PTSD-diagnosed individuals. Their sample of 51 women who had experienced interpersonal violence also self-reported experiencing insomnia and global sleep impairment, including reduced sleep time, more night-time awakenings, and longer sleep onset latency.

These subjective reports of PTSD-associated sleep disturbance are, however, not consistent with objective reports gathered by polysomnographic and actigraphy studies of individuals with the disorder. Furthermore, there is marked inconsistency within this latter group of studies. For instance, whereas some report no evidence of objectively-measured sleep disruption in PTSD (see, e.g., Hurwitz et al., 1998; Engdahl et al., 2000; Klein et al., 2002), others confirm that the disorder is characterized by gross sleep architecture disruptions, such as increased sleep latency (amount of time taken to fall asleep), decreased sleep efficiency (percentage of time spent asleep vs. time in bed), increased number of awakenings, and more time spent awake after sleep onset (Mellman et al., 1995a,b, 1997; Calhoun et al., 2007; Capaldi et al., 2011). Meta-analytic reviews of studies focusing more closely on disruptions to specific sleep stages suggest that, relative to sleep in individuals without PTSD, sleep in PTSD-diagnosed individuals is characterized by decreased sleep depth [e.g., increased stage 1 sleep, decreased slow-wave sleep (SWS)], increased REM pressure (e.g., shorter latency to enter rapid eye movement sleep, increased density of REM sleep), and disrupted sleep continuity (e.g., increased number of awakenings) (Kobayashi et al., 2007; Baglioni et al., 2016).

In summary, whereas historical accounts and subjective reports suggest that sleep disturbances are pervasive, ubiquitous, and of major consequence in PTSD, studies using objective measures present a different picture, one characterized by variable manifestation of such symptoms and relatively small effect sizes (indicating relatively mild instability) when sleep disturbances are detected. As Lavie (2001, p. 1830) states, “subjective reports are out of proportion to the frequency and severity of objective sleep-laboratory findings.”

The question of why these two sources of data are so out of kilter with one another has been addressed by several recent studies. One proposal rests on the concept of sleep-state misperception (Hurwitz et al., 1998; Lavie, 2001; Calhoun et al., 2007), which suggests that individuals with PTSD misperceive the true characteristics of their sleep disturbance (specifically, they overestimate the extent of the disruptions they experience during sleep, reporting far less sleep than they objectively achieve and far more night-time awakenings than are objectively measured). For example, Hurwitz et al. (1998) demonstrated that PTSD-diagnosed combat veterans underestimated their total sleep time, and reported distressing sleep disruption in the absence of objective confirmation, via polysomnography, of any clinically significant sleep disorder. Extending these findings, Dagan et al. (1997) and Calhoun et al. (2007) showed that there were non-significant correlations between actigraphic data (which suggested no more sleep disruption than in healthy controls) and subjective reports (which contained consistent complaints of poor sleep). Hence, this set of studies suggests that PTSD-diagnosed individuals not only overestimate the extent to which their sleep is disrupted, but are also unaware of the actual variability in their sleep patterns.

Some important issues remain to be clarified in this literature on sleep-state misperception, however. One relates to the question of whether this phenomenon is specific to PTSD, or even to psychiatrically disordered individuals in general. A number of studies, including some of those mentioned above, document the presence of sleep-state misperception in healthy individuals (see, e.g., Hurwitz et al., 1998; Calhoun et al., 2007), but few published studies have examined just how common the phenomenon might be in the general population. Another issue relates to methodological inconsistency. Whereas some studies use polysomnography to capture details of sleep architecture, others use the relatively cruder actigraphy technology. Similarly, in terms of subjective measures, no instrument is used consistently across studies. Relatedly, some studies (e.g., Woodward et al., 1996) correlate findings from retrospectively gathered subjective data (e.g., from the Pittsburgh Sleep Quality Index (PSQI), which asks about sleep over the previous month) with those from 1 to 2 nights of objectively measured sleep, whereas others (e.g., Klein et al., 2003) correlate findings from sleep diaries (filled in the morning after a night of sleep in either the home or the laboratory) with objective data from the same night of sleep.

Hence, there remain numerous questions about the nature, and even the existence, of sleep-state misperception in PTSD. Only a handful of studies have attempted critical investigation of those questions. Of these, possibly the most methodologically sound was conducted by Kobayashi et al. (2012). They measured sleep quality using two objective measures (polysomnography, for laboratory sleep, and actigraphy, for home sleep) and two subjective measures (self-report questionnaire and sleep diary, both administered after laboratory sleep and home sleep) in PTSD-diagnosed individuals in comparison to trauma-exposed and healthy control participants. They found that PTSD-diagnosed individuals did not over-report their sleep disturbance. Instead, analyses of sleep diary and actigraphy data suggested that, in the home environment, these participants estimated their total sleep time more accurately than did participants in the other two groups. Similar analyses of sleep diary and PSG data suggested that, in the laboratory environment, all participants, regardless of group assignment, overestimated their sleep onset latency. Hence, Kobayashi and colleagues concluded that they did not find evidence for sleep state misperception in PTSD-diagnosed individuals, and speculated that perhaps they were more vigilant in their home environment, and therefore more accurate at estimating their sleep duration, than they were in the laboratory (i.e., they felt relatively safer in the laboratory than at home).

Few studies outside of Kobayashi et al. (2012) assess whether PTSD-diagnosed participants' subjective reports of their sleep quality, under conditions of objective measurement, are representative of their general sleep quality, and hence of their everyday experience of sleep disruption. In other words, laboratory-based studies of sleep in PTSD are, generally, governed by the assumption that the objective measures they take are representative of everyday sleep quality. Without testing that assumption, these studies accept that their objective measures should demonstrate an equivalent degree of sleep disruption as is consistently reported by a variety of everyday subjective sleep quality measures. When such equivalence is not demonstrated, explanatory mechanisms such as sleep-state misperception are employed. Here, we test a simpler proposition: Sleep in the laboratory is not the same as sleep at home, so that inconsistency between measures of objective and subjective sleep quality rests on the false assumption of equivalent environmental context (and is not, for instance, due to individual misperception).

## Methods

### Study design

This investigation, which was part of a larger study investigating relations between cognition, affect, and sleep in PTSD-diagnosed individuals, featured a cross-sectional quasi-experimental design. Group membership was the independent variable, and measures of subjective sleep (as captured by self-report questionnaires) and objective sleep (as captured by polysomnography) were the outcome variables.

### Participants

Participants were recruited from three branches of the Rape Crisis Cape Town Trust, each located in a low socioeconomic status community, and through advertisements placed in local newspapers distributed to those areas. With regard to individuals recruited in the former way, we worked closely with counselors at the Rape Crisis center to identify potential participants.

**Figure 1:**
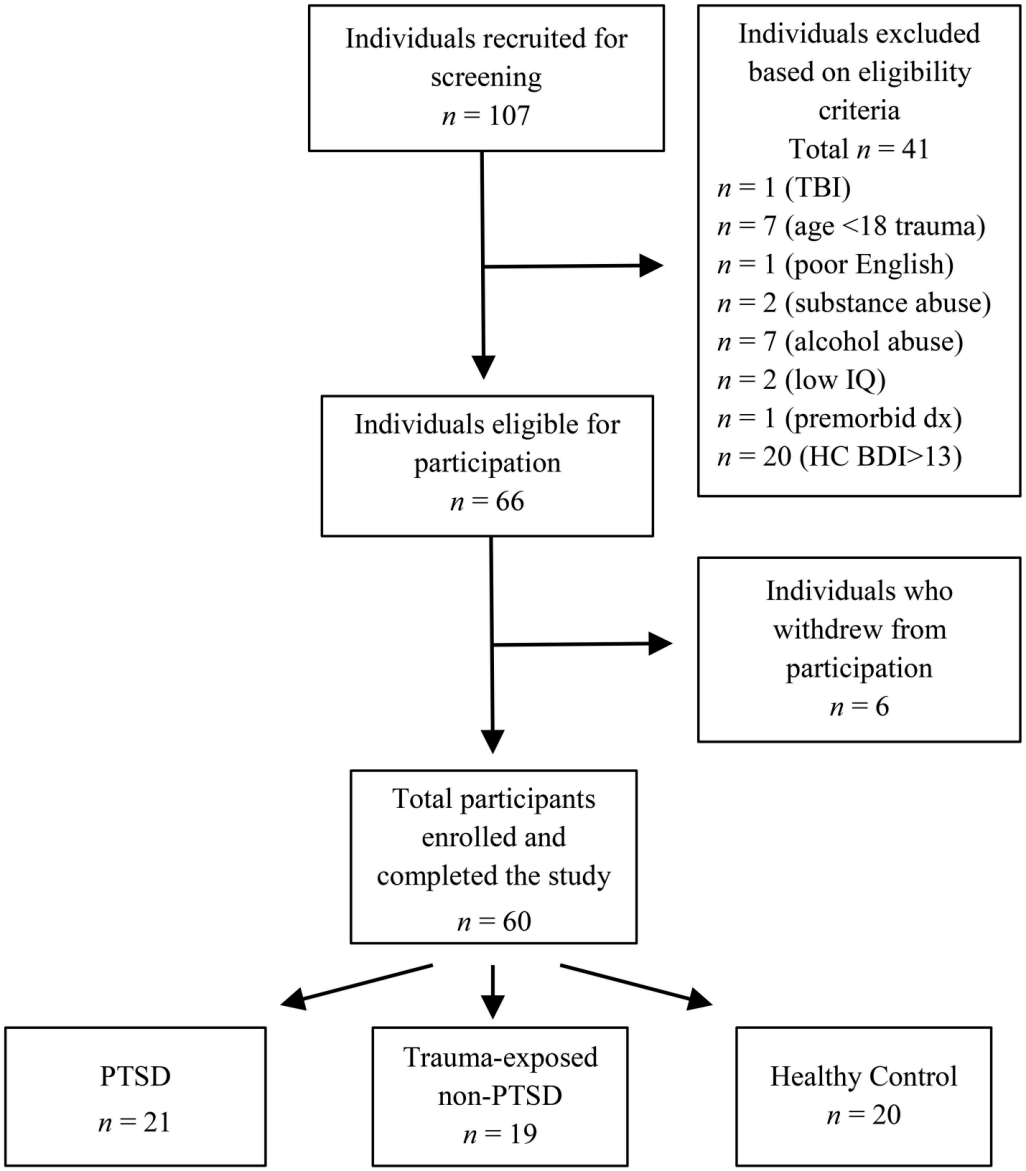
Recruitment of participants. TBI, traumatic brain injury; age <18 trauma, sexual assault at age 17 years or younger; premorbid dx, premorbid psychiatric diagnosis; Low IQ, Performance IQ more than 1.5 SD below the sample mean; HC BDI>13, healthy control participants with Beck Depression Inventory—Second Edition score >13.

Overall, we recruited 107 women for screening. Based on the eligibility criteria outlined below, 66 met the criteria for inclusion and were invited to the next stage of the study. However, six withdrew from the study after screening, due to work or other commitments, leaving a final sample of 60 participants. Figure 1 outlines the recruitment flow.

Each participant was included in one of three groups based on the eligibility criteria: PTSD (*n* = 21), trauma-exposed non-PTSD (TE; *n* = 19), and healthy control (HC; *n* = 20). Groups were matched in terms of age, level of education, IQ, income level, employment status, language, and smoking status. Smokers were included in the sample. Although studies show that smoking influences sleep (Zhang et al., 2006), the eligibility criteria described below were already restrictive, with a large number of potential participants excluded from the study. To ensure the recruitment of an adequate sample, smokers were permitted to participate provided no statistically significant differences in smoking status existed between PTSD, TE, and HC groups. Furthermore, the actual differences between smokers and non-smokers are relatively small: For example, smokers take ~5 min longer to fall asleep, experience 14 min less sleep, and have ~6% less SWS than non-smokers (Zhang et al., 2006).

We chose to only recruit female survivors of sexual assault because most PTSD studies recruit male war veterans and several authors have highlighted that studies recruiting female participants with trauma etiologies other than war are important to understand the whole spectrum of the disorder (Santiago et al., 2013; Hall Brown et al., 2015). Furthermore, we included the TE group to differentiate between the experience of trauma and a diagnosis of PTSD. Only a small percentage of individuals who experience a traumatic event go on to develop PTSD (Kessler et al., 1995; Kaminer et al., 2008). From this perspective, it is important to differentiate between those who develop psychopathology in the aftermath of trauma and those who do not.

#### Eligibility criteria

Our primary inclusion criteria were psychiatric status and English fluency. We excluded potential participants diagnosed with any DSM-IV (APA, 2000) Axis I disorders (except PTSD), although individuals in the PTSD and TE groups who presented with other anxiety and mood disorders secondary to the trauma remained eligible. We excluded potential participants who were not fluent in English because several of our measures were only available in that language.

Furthermore, we enforced the following criteria strictly, because each factor listed below has an independent effect on sleep quality (Myers and Badia, 1995; Stewart et al., 1998; Harvey et al., 2003; Vermetten et al., 2003; Grigg-Damberger, 2012; Mellman et al., 2014). We excluded from participation any individual (a) with a 1-year history of alcohol or other substance abuse, (b) below the age of 18 years and above the age of 40 years, (c) who at the time of recruitment was taking sedative medication to regulate sleeping patterns, or who was prescribed psychoactive medication (including antidepressants), (d) who had experienced trauma more than 5 years or fewer than 6 months prior to screening, or (e) who carried neurological conditions (e.g., epilepsy, traumatic brain injury).

### Materials and apparatus

#### Diagnostic and screening instruments

The *Mini International Neuropsychiatric Interview* (MINI version 5.0.0; Sheehan et al., 1998) is a well-validated structured diagnostic interview that assesses the major DSM-IV Axis I psychiatric disorders. South African studies have used the MINI in a variety of community research settings (e.g., Seedat et al., 2007; Onah et al., 2016). We used it to confirm diagnoses of PTSD, and to exclude the presence of other DSM-IV Axis I psychiatric conditions across all groups, with the exception of anxiety and mood disorders secondary to the trauma in the PTSD and TE groups. The MINI was also the primary instrument used to determine selection of the healthy control group: These participants were required to carry no MINI-assessed psychiatric diagnoses, and were screened carefully to ensure that they had not experienced anything that qualified as a DSM-IV-TR PTSD Criterion A traumatic event.

The *Clinician Administered PTSD Scale* (CAPS; Blake et al., 1995) is a structured interview designed to assess for the presence of core and associated PTSD symptoms. It has been used in several South African studies (e.g., Martenyi et al., 2002; Suliman et al., 2015). We used the CAPS to validate the PTSD diagnosis provided initially by the MINI.

The *Beck Depression Inventory—Second Edition* (BDI-II; Beck et al., 1996) is a standardized 21-item self-report questionnaire that assesses current presence and severity of depression in adults. In South Africa, as in other countries around the world, the BDI-II is used in clinical settings and as a research tool (e.g., Berard et al., 1998; Stellenberg and Abrahams, 2015). We used it to help provide information about the level of depression reported by participants in the PTSD and TE groups. Potential participants in the HC group with a BDI score ≥14 were excluded.

#### Measures of subjective sleep quality

The *Pittsburgh Sleep Quality Index* (PSQI; Buysse et al., 1989) is a brief (10 min) 19-item self-report questionnaire. In South Africa, as in other countries around the world, it is used in both clinical and research settings (Henry et al., 2015). We used the PSQI to assess self-reported sleep quality over the month prior to laboratory testing.

We also created an adaptation of the standard PSQI (the Laboratory PSQI) that allowed us to assess subjective sleep quality in the laboratory. Hence, we could compare participants' subjective sleep quality over the past month to their sleep quality in the laboratory. We used this approach, rather than conventional sleep diaries, because both the original PSQI and the Laboratory PSQI calculate a sleep quality score.

Furthermore, we asked two additional questions related to subjective sleep quality in the laboratory: “*Did the equipment bother you?*” with options on a 4-point scale (0 = not at all; 1 = a little; 2 = quite a lot; 3 = a lot) and “*How did you sleep in the lab in comparison with your home?*” with options on a 4-point scale (0 = better; 1 = same; 2 = little worse; 3 = worse). We also asked participants to reflect on their laboratory sleep quality in comparison with their normal sleep at home, and noted their responses verbatim.

#### Sleep laboratory equipment

We completed polysomnographic (PSG) recordings of sleep in our laboratory, which is equipped with two 16-channel Nihon Kohden NeuroFax EEG9000 electroencephalographs (EEG) adapted for sleep research. This equipment maps out sleep architecture and consists of EEG electrodes that measure brain activity, electrooculograph (EOG) electrodes that monitor eye movements, electromyograph (EMG) electrodes that measure muscle tone, and electrocardiograph (ECG) electrodes that measure heartbeat. These different measures are essential in identifying REM sleep, as it is not always reliably identified through brain activity measures alone (Keenan, 2009).

We used a bipolar longitudinal montage, including the bipolar derivations F3-C3, C3-P3, P3-O1, and F4-C4, C4-P4, P4-O2 in combination with a referential montage using F3-A2, C3-A2, O1-A2, and F4-A1, C4-A1, O2-A1 derivations. We used a combination approach to ensure the integrity of all records. Referential montages provide excellent EEG resolution, but are susceptible to signal loss if one of the referential electrodes (A1 or A2) fails. Bipolar derivations, in contrast, do not provide as good a resolution, but are less dependent on specific electrodes. All electrodes were placed according to the international 10–20 system. Standardized filters for recording sleep were employed for the EEG and EOG (0.5–35 Hz), EMG (10–70 Hz), and ECG (1–70 Hz) leads to ensure integrity of the signal. The ground electrode was placed on the middle of the forehead.

### Procedure

The procedure included an initial screening, followed by two nights at the sleep laboratory. The screening took place in a private, quiet room in the UCT Department of Psychology. Where necessary, transport was provided for participants.

#### Screening

Each participant read and signed a detailed informed consent document, after which the screening measures listed above were administered. When screening potential PTSD and TE participants we also took a careful clinical history, alongside the screening measures, to ensure that other psychopathologies (such as panic disorder) were secondary to the traumatic experience and did not precede the trauma.

At the conclusion of these screening and diagnostic procedures, we debriefed the participant about the study procedures up to that point. If the participant was deemed suitable for continued enrolment, we scheduled an appointment for the two sleep nights, and assigned the participant to one of the three groups.

#### Sleep testing

Participants attended an adaptation night prior to the single night of sleep that was recorded for statistical analysis. The adaptation night, which consisted of a full 8-h period of sleep in the laboratory and included the placement of electrodes, served to habituate participants to the laboratory setting.

Participants were asked to refrain from daytime naps, excessive exercise, and alcohol in the day prior to sleep testing. They were also asked to only ingest drinks containing caffeine (coffee, Coke, energy drinks) first thing in the morning and not during the rest of the day. Smoking participants were allowed to smoke conservatively (at or below their normal intake), because studies show that acute withdrawal is worse for sleep than continued smoking (Zhang et al., 2006; Jaehne et al., 2015).

Participants arrived at the UCT Sleep Sciences laboratory ~2 h before their bedtime on the adaptation night and ~3 h before their bedtime on the experimental night. Upon arrival on the adaptation night, each participant was briefed about the procedures for the evening and the morning. Participants were shown their rooms and provided with details about their environment, such as the use of the bathroom as well as emergency procedures should they require any assistance during the night.

Thereafter, we prepared each participant for a night's sleep while attached to the polysomnograph. More specifically, we attached the EEG electrodes to the head using EC2 paste, and the EOG, EMG, and ECG electrodes to the face and chest using stickers designed as electrodes. Once the sleep equipment was set up, we tested that all the channels were working correctly by asking the participant to perform simple actions such as blinking and biting. We recorded the impedance to ensure that it was below 5 O for all channels. Participants were also reassured that they could sleep in their normal body positions. Participants were then allowed an 8-h period of sleep, commencing within 30 min of their regular bed-time. After 8 h, they were woken and all the PSG equipment was removed.

On the experimental night, participants completed tasks, and measures related to the larger study immediately upon arrival at the laboratory. Thereafter, the procedure was identical to that of the adaptation night.

In the morning after the experimental night, participants were fully debriefed about the study procedures. Each participant was shown her sleeping patterns, with explanations about the various sleep stages. Where appropriate, we briefed the participant about best practice regarding sleep hygiene. Each participant was also remunerated ZAR300 (at the time of the study, ~US$22).

This study was carried out in accordance with the recommendations of the Research Ethics Committees of the University of Cape Town's Department of Psychology and Faculty of Health Sciences. All subjects gave written informed consent in accordance with the Declaration of Helsinki.

### Statistical analysis

The data were scored by two independent judges according to the criteria based on Rechtschaffen and Kales (1968). Both judges were blind to the group allocation of each participant. Inter-rater reliability ranged from 0.85 to 0.95. In addition to the standard scoring of sleep stages and arousals, arousals specifically arising during REM sleep were manually recorded. These arousals were recorded in two separate categories. First, all REM arousals that met the standard criteria were recorded. Second, REM arousals that met the standard criteria, but resulted in NREM1 or waking, were recorded.

Inferential statistical analyses were completed using SPSS version 23, with α set at 0.05 for all decisions regarding statistical significance. We first examined the data for deviations from normality and variance of the distribution. Unless there were substantial deviations with regard to both distributional characteristics, we used parametric analytic techniques because, for example, ANOVA is robust to violations of its assumptions (Spencer et al., 2017).

Before testing whether PTSD-diagnosed individuals had significantly more sleep disruption than TE and HC participants, we checked to see whether there were any significant between-group differences in bedtime. Such differences may reflect circadian variance between participants, which may independently influence sleep quality. A one-way ANOVA detected no significant between-group differences in self-reported bedtime, *F*_(2, 57)_ = 0.54, *p* = 0.587. Circadian variation, therefore, did not need to be accounted for in subsequent analyses.

#### Between-group differences: subjective sleep quality

The PSQI and the Laboratory PSQI characterized participants' subjective sleep quality. Whereas the PSQI assessed participants' reports of their sleep quality over the month immediately prior to responding, the Laboratory PSQI assessed their sleep quality during the laboratory night. We conducted two sets of one-way ANOVAs to determine whether (a) PTSD-diagnosed individuals, in comparison with TE and HC participants, report poor everyday subjective sleep quality, and (b) whether this pattern of self-reported sleep disruption was replicated in the sleep laboratory environment. We followed significant omnibus ANOVA results with orthogonal planned comparisons to test where significant group differences lay. Then, within each group, we compared PSQI and Laboratory PSQI scores using a paired-sample *t*-test. Finally, we used one-way ANOVA to examine between-group differences in participant responses to the two subjective sleep quality questions.

#### Between-group differences: objective sleep quality

To evaluate the PSG data, we conducted separate one-way ANOVAs examining between-group differences on the measures of sleep latency, sleep efficiency, number of awakenings (defined as a period longer than 1.5 min after sleep onset), number of spontaneous arousals (defined as a period of abrupt EEG shift during the night, usually an increase in EEG frequency, lasting at least three or more seconds), number of minutes spent awake after sleep onset, NREM1 percentage, NREM2 percentage, SWS percentage, REM percentage, REM latency, REM arousals, and REM arousals leading to NREM1 or waking. We also analyzed the data for a composite variable combining NREM1 percentage and SWS percentage, based on the findings by Kobayashi et al. (2007) that NREM1 and SWS disruptions are the most prominent objective sleep-stage deficits in PTSD-diagnosed individuals. To create this composite variable, we transformed each participant's NREM1 percentage and SWS percentage into a *z*-score, using the mean and standard deviation for each variable. Because sleep disruption is marked by *increased* NREM1 percentage and *decreased* SWS percentage, we multiplied all *z*-transformed NREM1 values by −1 so that *z*-transformed NREM1 and SWS scores could be combined. We then averaged the *z*-score calculated for NREM1 percentage and SWS percentage to form the NREM1/SWS composite for each participant.

As before, we followed significant omnibus ANOVA results with orthogonal planned comparisons to test where significant group differences lay.

#### Relationship between subjective and objective sleep quality

Using Spearman's rho correlation coefficient, we described the magnitude of association between (a) PSQI scores and PSG outcome variables, and (b) Laboratory PSQI scores and PSG outcome variables. We ran these correlational analyses for the entire sample, and for each group separately.

## Results

### Sample characteristics

Table 1 presents the sample's sociodemographic characteristics. Data from the MINI, BDI, CAPS, and clinical interview were used to characterize the psychiatric conditions, symptom presentation, counseling attendance record, and symptom severity in the PTSD and TE groups. Participants in the HC group were, by definition, free of any psychiatric disorders and free of experiences that would meet the characteristics of a DSM-IV-TR Criterion A traumatic event. Table 2 describes the psychiatric characteristics of the sample.

**Table 1:** Sociodemographic characteristics of the current sample (*N* = 60).

**Variable**	**Group**			***F*/*χ^2^***	***p***
	**PTSD (*n* = 21)**	**TE (*n* = 19)**	**HC (*n* = 20)**		
Age	25.54 (4.35)	24.42 (4.53)	25.30 (4.62)	0.33	0.72
Education^a^	11.67 (1.59)	12.53 (2.09)	12.90 (2.00)	2.28	0.11
Income^b^				12.42	0.10
0-R999	1	1	1		
1,000-R2,499	3	6	5		
2,500-R5,499	12	7	5		
5,500-R9,999	0	4	5		
10,000+	5	1	4		
Employment status				6.68	0.15
Unemployed	7	8	10		
Employed	11	6	3		
Student	3	5	7		
Smoking status				5.01	0.09
Yes	7	1	5		
No	14	18	15		

*TE, Trauma Exposed; HC, Healthy Control. For the variables Age and Education, means are presented with standard deviations in parentheses. For the variables Income, Employment Status, and Smoking Status, Fisher's exact test statistic is presented*.

a *Number of years of completed education*.

b *In South African Rands (ZAR). At the time of study, the ZAR:US$ exchange rate was 11.45*.

**Table 2:** Psychiatric characteristics of the current sample (*N* = 60).

**Variable**	**Group**			***F*/*t*/*χ^2^***	***p***
	**PTSD (*n* = 21)**	**TE (*n* = 19)**	**HC (*n* = 20)**		
BDI-II score	30.14 (6.26)	17.72 (7.98)	6.20 (3.41)	78.86	<0.001^***^
Contrast 1^a^				10.55	<0.001^***^
Contrast 2^b^				5.81	<0.001^***^
CAPS total score	67.67 (14.12)	28.95 (11.69)		9.39	<0.001^***^
Time since trauma^c^	1.30 (1.02)	1.21 (1.07)		0.28	0.78
Counseling sessions^d^	3.77 (2.32)	4.20 (3.49)		−0.38	0.71
Number of MINI dx^e^	2.10 (1.22)	1.37 (1.67)		1.58	0.12
Current counseling				3.48	0.09
Yes	9	3			
No	12	16			

*Means are presented with standard deviations in parentheses. TE, Trauma-Exposed; HC, Healthy Control; BDI-II, Beck Depression Inventory-Second Edition; CAPS, Clinician-Administered PTSD Scale*.

a *Compares the PTSD group to the combined TE and HC groups*.

b *Compares the TE and HC groups*.

c *Time, in years, between trauma exposure and study participation*.

d *Total number of counseling sessions attended by participants since trauma*.

e *Number of Mini International Neuropsychiatric Inventory diagnoses*.

*** *p < 0.001*.

A few aspects of Table 2 are worth mentioning. Although participants in the PTSD group had significantly greater PTSD-related symptom severity than those in the TE group (as expected, given the manner in which group assignment was conducted), these two groups did not differ significantly with regard to other clinical characteristics, aside from depressive symptomatology.

A one-way ANOVA detected statistically significant between-group differences with regard to BDI-II scores. Consistent with previous research showing that more severe trauma symptoms are associated with greater depression (Weathers et al., 2001), and with the study's selection criteria, a set of *post-hoc* planned orthogonal contrasts suggested that, on average, (a) participants in the PTSD group reported significantly more depressive symptoms than those in the other two groups, and (b) participants in the TE group reported significantly more depressive symptoms than those in the HC group. In fact, HC participants indicated no presence of depression.

A careful patient history and administration of the MINI revealed that most participants in the PTSD and TE groups had other DSM-IV-TR mood- and anxiety-related psychiatric diagnoses secondary to the trauma experience. Furthermore, participants in the trauma groups had experienced only one event of sexual assault, which was considered the index trauma.

#### Between-group differences: subjective sleep quality

Table 3 presents the results of the analyses examining between-group differences for the PSQI and Laboratory PSQI.

**Table 3:** Subjective sleep quality: descriptive statistics and between-group comparisons (*N* = 60).

**Variable**	**Group**			***F /t***	***p***	**ESE**
	**PTSD (*n* = 21)**	**TE (*n* = 19)**	**HC (*n* = 20)**			
PSQI^a^	9.65 (4.18)	5.88 (3.00)	4.16 (2.65)	13.54	<0.001^***^	0.34
Contrast 1^b^				4.92	<0.001^***^	1.38
Contrast 2^c^				1.53	0.13	0.61
Laboratory PSQI	5.29 (3.07)	5.26 (3.83)	4.05 (1.96)	1.09	0.34	0.04

*Means are presented with standard deviations in parentheses. TE, Trauma-Exposed; HC, Healthy Control; ESE, effect size estimate (in this case, η^2^ for F statistic and Cohen's d for t statistic)*.

a *Due to participant error in completing questionnaires, n = 20 for PTSD, n = 17 for TE, and n = 19 for HC*.

b *Compares the PTSD group to the combined TE and HC groups*.

c *Compares the TE group to the HC group*.

*** *p < 0.001*.

Regarding PSQI scores, the main analysis detected significant between-group differences. A series of orthogonal contrast analyses suggested that, on average, (a) PTSD-diagnosed individuals reported poorer everyday sleep quality over the previous month in comparison with the combined TE and HC groups, and (b) there was no significant difference in everyday subjective sleep quality between the TE and HC groups.

A PSQI score >5 indicates clinically significant subjective sleep disruption. All but one participant in the PTSD group had a score exceeding this cut-off score. In the TE group, approximately equal numbers of participants fell above and below the cut-off score (PSQI >= 5, *n* = 10; PSQI < 5, *n* = 7). In the HC group, most participants experienced no sleep disruption (PSQI < 5, *n* = 15 participants); only a small minority reported experiencing mild sleep disruption (PSQI = 5 or 6, *n* = 4).

Regarding Laboratory PSQI scores, the main analysis detected no significant between-group differences. This result suggests that the usual pattern of subjective sleep disruption reported by PTSD-diagnosed individuals was not replicated in the laboratory.

Regarding within-group comparisons of PSQI and Laboratory PSQI scores, analyses detected no significant differences in both the TE and the HC groups, *t*_(16)_ = 0.60, *p* = 0.56, and *t*_(18)_ = 0.23, *p* = 0.82, respectively. However, PTSD-diagnosed participants had significantly lower Laboratory PSQI scores than PSQI scores, *t*_(19)_ = 4.06, *p* = 0.001 (*M*_Laboratory PSQI_ = 5.29, *SD*_Laboratory PSQI_ = 3.05; *M*_PSQI_ = 9.65, *SD*_Laboratory PSQI_ = 4.18).

These results suggest that, whereas for TE and HC participants the reports regarding home and laboratory sleep quality were similar, PTSD-diagnosed participants reported better sleep quality in the laboratory than at home.

Regarding participants' responses to the question *Did the equipment bother you?* analyses detected no significant between-group differences, *F*_(2, 57)_ = 0.20, *p* = 0.82. This result suggests that, on average, participants across the three groups rated the contribution of the equipment to their sleep quality similarly. Furthermore, the modal response indicated that participants felt that the equipment did not bother them at all (*n* = 52: equipment did not bother them at all; *n* = 8: equipment bothered them a little).

Regarding participants' responses to the question *How did you sleep in the lab in comparison with your home?* analyses also detected no significant between-group differences, *F*_(2, 54)_ = 2.09, *p* = 0.13. On average, participants across the three groups rated their home and laboratory sleep quality similarly. Furthermore, the modal rating indicated that approximately equal numbers of participants felt that they slept either better or the same in the laboratory as in their home environment (*n* = 26: slept better in the laboratory; *n* = 25: slept the same in the laboratory as at home; *n* = 6: slept a little worse in the laboratory).

Finally, Table 4 lists qualitative responses to the question asking for reflection on differences or similarities between home and laboratory sleep quality. The most common response, in particular for PTSD-diagnosed individuals, was the feeling that the sleep laboratory environment was quieter, safer, more comfortable, and freer from distractions than the home environment.

**Table 4:** Participant qualitative responses regarding the difference in sleep quality between the laboratory and the home environment (*N* = 60).

**Participant number**	**Group**	**Verbatim participant response defining sleep quality difference between laboratory and home environment**
2	PTSD	–
3	PTSD	–
5	PTSD	–
7	PTSD	Cool and quiet
8	PTSD	Felt safe
9	PTSD	–
10	PTSD	Quiet; safe
13	PTSD	Told herself she has to sleep
15	PTSD	Not used to sleeping here
16	PTSD	I don't know, woke up many times
18	PTSD	Felt the same as at home
20	PTSD	Comfortable
24	PTSD	No noise–don't hear gun shots, taxi, it's just quiet
26	PTSD	–
27	PTSD	Comfortable
48	PTSD	Relaxed, alone in the bed—at my house I sleep with my sisters and brothers
52	PTSD	Used to surroundings from the previous night and nice and quiet
54	PTSD	It's quiet
57	PTSD	I don't know, feels comfortable
58	PTSD	Quiet
60	PTSD	Because of environment–quiet, no disturbances
1	TE	Not used to it; perhaps food poisoning on wed added to sleep difficulties
4	TE	Quiet here, didn't play with the phone
6	TE	The same as usual
11	TE	Always sleeps the same
12	TE	Not the dream that woke me; quiet here; no distractions
14	TE	Was sleepy but took a long time to fall asleep
17	TE	Felt the same comfortable as home
19	TE	Used to the lab now
22	TE	Didn't think much; felt safe
29	TE	Nice and quiet
44	TE	Because its comfortable
47	TE	Thinking about children
49	TE	No bad dreams–comfortable
50	TE	So comfortable and quiet
51	TE	Felt like she slept in comparison to the adaptation night
53	TE	Nothing new happened; slept like usually does
55	TE	Quiet here, no dog chasing something
56	TE	Don't know
59	TE	–
21	HC	Fell asleep quick; safe; no worries
23	HC	–
25	HC	Maybe because I am comfortable
28	HC	Because I was so tired
30	HC	Because of the comfortable bed
31	HC	–
32	HC	Quiet and no TV
33	HC	Whole experience, being warm, quiet, same as at home
34	HC	Wake up often at home because of children
35	HC	Bed is comfortable
36	HC	No one to bother, no noise
37	HC	More used to the environment
38	HC	Nothing bothered me; very quiet; went straight to sleep
39	HC	Used to the lab. Not used to sleeping on her own–usually sleep with child
40	HC	Comfortable
41	HC	Didn't feel uncomfortable
42	HC	When not doing any projects at varsity she sleeps well
43	HC	Comfortable
45	HC	My second day so getting used to it
46	HC	Nothing happened and I slept as usual as at home

#### Between-group differences: objective sleep quality

Table 5 shows the results of the series of analyses examining between-group differences in objective sleep quality.

**Table 5:** Objective sleep quality: descriptive statistics and between-group comparisons (*N* = 60).

**Variable**	**Group**			***F/t***	***p***	**ESE**
	**PTSD (*n* = 21)**	**TE (*n* = 19)**	**HC (*n* = 20)**			
Sleep latency	27.93 (34.44)	15.18 (11.87)	26.75 (33.41)	1.16	0.32	0.04
Sleep efficiency	84.79 (14.57)	90.87 (5.69)	87.55 (7.65)	1.77	0.18	0.06
Awakenings	3.57 (3.17)	2.05 (1.62)	3.45 (2.48)	2.17	0.12	0.07
Arousals	126.76 (63.61)	107.16 (33.54)	111.55 (31.39)	1.02	0.37	0.03
WASO	45.40 (58.55)	30.55 (23.26)	34.63 (23.01)	0.77	0.47	0.03
NREM1%	16.85 (7.08)	13.91 (4.01)	12.30 (5.32)	3.40	0.04^*^	0.11
Contrast 1^a^				2.44	0.02^*^	0.67
Contrast 2^b^				0.88	0.38	0.34
NREM2%	56.24 (8.52)	57.20 (5.83)	53.74 (6.33)	1.27	0.29	0.04
SWS%	8.90 (8.62)	9.27 (8.05)	14.66 (7.30)	3.25	0.04^*^	0.10
Contrast 1^c^				−2.54	0.01^*^	0.70
Contrast 2^d^				−0.15	0.88	−0.04
REM%	17.91 (5.97)	19.63 (4.22)	19.31 (4.30)	0.71	0.50	0.02
REM latency	90.10 (31.13)	97.37 (58.56)	104.60 (42.61)	0.51	0.60	0.02
REM arousals	20.20 (8.56)	20.68 (11.07)	21.35 (9.60)	0.07	0.93	0.002
REM 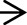 NREM1/W	10.75 (4.33)	10.53 (6.78)	8.55 (4.45)	1.05	0.36	0.04
NREM1% + SWS%	−0.32 (0.84)	−0.08 (0.67)	0.41 (0.78)	4.81	0.01^*^	0.14
Contrast 1^a^				−2.36	0.02^*^	−0.63
Contrast 2^a^				−2.10	0.04^*^	−0.67

*Means are presented with standard deviations in parentheses. Degrees of freedom were (2, 57) for all variables except REM Latency, REM Arousals and REM 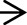 NREM1/W, where there were (2, 56) degrees of freedom because 1 participant in the PTSD group did not achieve REM sleep. TE, Trauma-Exposed; HC, Healthy Control; ESE, effect size estimate (in this case, η2 for F statistic and Cohen's d for t statistic); Awakenings, number of awakenings after sleep onset; Arousals, number of arousals after sleep onset; WASO, number of minutes spent awake after sleep onset; NREM1%, NREM1 percentage; NREM2%, NREM2 percentage; SWS%, SWS percentage; REM%, REM percentage; REM 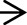 NREM1/W, REM arousals leading to NREM1 or wake; NREM1% + SWS%, composite of NREM1 percentage and SWS percentage*.

a *Compares the PTSD group to the combined TE and HC groups*.

b *Compares the TE group to the HC group*.

c *Compares the HC group to the combined TE and PTSD groups*.

d *Compares the TE group to the PTSD group*.

* *p < 0.05*.

The analyses detected few significant between-group differences. NREM1 percentage, SWS percentage, and the NREM1/SWS composite reached omnibus ANOVA significance. Follow-up planned comparisons of the NREM1 percentage data revealed that, on average, (a) participants in the PTSD group experienced a significantly greater amount than those in TE and HC groups combined, and (b) there was no significant difference between the TE and HC groups. Regarding SWS percentage, planned comparisons showed that, on average, (a) the PTSD and TE groups combined had a significantly lower amount than the HC group, and (b) there was no significant difference between the PTSD and TE groups. Regarding the NREM1/SWS composite variable, planned comparisons revealed that, on average, (a) participants in the PTSD group experienced a combination of greater NREM1-and lower SWS-percentage than those in the TE and HC groups combined, and (b) participants in the TE group experienced participants recorded higher values than did those in the HC group. These differences were associated with effect sizes of moderate magnitude.

#### Relationship between subjective and objective sleep quality

Table 6 presents the correlation matrices. Regarding associations between PSQI scores and PSG outcome variables across the entire sample, analyses detected only one significant correlation (i.e., a negative relationship between everyday subjective sleep quality and REM percentage).

**Table 6:** Correlation Matrix: associations (Spearman's rho) between subjective and objective sleep measures (*N* = 60).

**Objective sleep variable**					**Group**											
	**Entire sample **(*****N*** = **60)****				**PTSD **(*****n*** = **21)****				**TE **(*****n*** = **19)****				**HC **(*****n*** = **20)****			
	**PSQI**		**Lab PSQI**		**PSQI**		**Lab PSQI**		**PSQI**		**Lab PSQI**		**PSQI**		**Lab PSQI**	
	***rho***	***p***	***rho***	***p***	***rho***	***p***	***rho***	***p***	***rho***	***p***	***rho***	***p***	***rho***	***p***	***rho***	***p***
Sleep latency	−0.02	0.88	0.22	0.08	−0.58	0.81	0.22	0.34	0.05	0.86	0.12	0.62	−0.15	0.55	**0.54**	0.01^*^
Sleep efficiency	−0.08	0.54	−**0.43**	< 0.01^**^	0.04	0.88	−**0.53**	0.01^*^	−0.26	0.31	−0.44	0.06	0.16	0.53	−**0.53**	0.02^*^
Awakenings	0.05	0.72	0.25	0.06	0.17	0.48	**0.45**	0.04^*^	−0.09	0.74	0.19	0.45	−0.32	0.18	0.17	0.48
Arousals	0.01	0.94	−0.05	0.70	−0.13	0.59	−0.17	0.47	−0.34	0.19	−0.01	0.99	0.04	0.88	0.12	0.61
WASO	0.11	0.41	**0.35**	< 0.01^**^	0.04	0.88	**0.49**	0.03^*^	0.25	0.33	0.39	0.10	−0.18	0.45	0.03	0.90
NREM1%	0.21	0.12	**0.32**	0.01^*^	0.18	0.44	0.35	0.12	−0.09	0.73	0.17	0.49	−0.26	0.28	0.44	0.06
NREM2%	−0.03	0.83	0.10	0.45	−0.29	0.22	0.16	0.49	0.12	0.66	−0.01	0.99	0.07	0.78	−0.18	0.94
SWS%	0.06	0.65	−0.17	0.20	0.44	0.06	−0.15	0.51	−0.06	0.83	−0.04	0.87	0.31	0.19	−0.19	0.41
REM%	−**0.32**	0.02^*^	−0.24	0.07	−0.42	0.07	−0.40	0.07	0.04	0.89	−0.09	0.73	−0.32	0.18	−0.19	0.42
REM latency	0.04	0.79	0.11	0.41	0.13	0.59	0.18	0.45	−0.18	0.50	0.10	0.68	**0.61**	<0.01^**^	0.16	0.51
REM arousal	−0.24	0.08	−0.19	0.14	−0.21	0.38	−0.28	0.23	−0.44	0.08	−0.23	0.34	−0.20	0.42	−0.01	0.99
REM 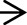 NREM1/W	−0.05	0.72	−0.08	0.54	0.10	0.69	−0.20	0.40	−0.39	0.12	−0.12	0.62	−0.35	0.15	0.01	0.97
NREM1% + SWS%	−0.09	0.53	−**0.28**	0.03^*^	0.15	0.53	−0.35	0.13	0.04	0.88	−0.06	0.81	0.33	0.17	−0.37	0.11

*For PSQI n = 20 for PTSD, n = 17 for TE, and n = 19 for HC due to participant error in completing the questionnaire. TE, Trauma-Exposed; HC, Healthy Control; PSQI, Pittsburgh Sleep Quality Index; Awakenings, number of awakenings after sleep onset; Arousals, number of arousals after sleep onset; WASO, number of minutes spent awake after sleep onset; NREM1%, NREM1 percentage; NREM2%, NREM2 percentage; SWS%, SWS percentage; REM%, REM percentage; REM 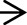 NREM1/W, REM arousals leading to NREM1 or wake; NREM1% + SWS%, composite of NREM1 percentage and SWS percentage. Numbers in boldface font indicate statistically significant results*.

* p < 0.05;

** *p < 0.01*.

Regarding associations between Laboratory PSQI scores and PSG outcome variables across the entire sample, analyses suggested that poorer laboratory subjective sleep quality was significantly and moderately associated with lower sleep efficiency, more awakenings, greater NREM 1 percentage, and a combination of lower SWS percentage and greater NREM 1 percentage. These results demonstrate that, for all participants, subjective reports about sleep in the laboratory were congruent with objective measures of sleep in the laboratory.

Regarding within-group associations between subjective and objective sleep measures, there was only one significant correlation between PSQI scores and PSG outcome variables (viz., in the HC group, PSQI scores were positively associated with REM latency). There were, however, a number of significant correlations between Laboratory PSQI scores and PSG outcome variables, particularly within the PTSD group. Within that group, poorer laboratory subjective sleep quality was associated with less sleep efficiency, more time spent awake after sleep onset, and more frequent awakenings (medium-to-large correlations).

Within the TE group, the analysis detected no significant correlations. Within the HC group, individuals with poorer laboratory subjective sleep quality took longer to fall asleep and had decreased sleep efficiency (large correlations).

## Discussion

PTSD-diagnosed individuals frequently self-report sleep disruption (see, e.g., Giosan et al., 2015; Werner et al., 2016). However, among studies using objective measures (e.g., polysomnography), there are relatively few, and inconsistent, reports of PTSD-related sleep disruption (see, e.g., Engdahl et al., 2000; Calhoun et al., 2007; Yetkin et al., 2010). To account for this discrepancy between subjective and objective studies, some authors have hypothesized that PTSD-diagnosed individuals have sleep-state misperception (i.e., that they self-report experiencing poor sleep quality, but objectively sleep relatively normally; Woodward et al., 1996; Lavie, 2001; Klein et al., 2003; Calhoun et al., 2007). We evaluated this sleep-state misperception hypothesis against the simpler proposition that, oftentimes, the environmental context of the sleep laboratory is so different from that of the home that sleep quality is quite different in one than in the other. In other words, we investigated whether the inconsistency between studies of objective and subjective sleep quality in PTSD arises from the fact that measures are taken from different environmental contexts (i.e., the laboratory and the home, respectively), contexts that hitherto have often been regarded as equivalent. We collected data on subjectively-reported sleep quality (in the home, and in the laboratory) and on objectively-measured, laboratory-based, sleep quality in PTSD-diagnosed participants from low-SES South African communities.

Our first step was to replicate circumstances under which sleep-state misperception might be proposed (in other words, to show that, when measuring sleep objectively in the laboratory PTSD-diagnosed individuals show relatively little disruption, but when measuring sleep subjectively in the home environment they report relatively major disruption). Indeed, regarding objective measures of sleep, analyses detected relatively little disruption in PTSD-diagnosed individuals. The analyses detected no between-group differences on any sleep variables other than NREM1 percentage and SWS percentage. Hence, in this sample it appears that, on average, PTSD-diagnosed individuals, relative to controls, took a similar amount of time to fall asleep, had a similar amount of sleep in comparison to time in bed, had a similar number of awakenings and arousals, had a similar percentage of NREM 2 and REM sleep, and experienced a similar number of arousals from REM sleep.

Collectively, these results suggest that those in the PTSD, TE, and HC groups experienced relatively similar patterns of sleep, when indexed by PSG measures. Where PTSD-diagnosed individuals did experience sleep disruptions, these were related to sleep-stage specific disturbances, rather than to gross disturbances in their sleep architecture. These findings are consistent with those reported by the two major meta-analyses of data in this field. Both Kobayashi et al. (2007) and Baglioni et al. (2016) found that sleep in PTSD is characterized by decreased sleep depth (i.e., a combination of increased NREM 1 percentage and decreased SWS percentage).

These results differ from numerous previous studies of PTSD-diagnosed individuals (e.g., Spoormaker and Montgomery, 2008) in that they showed no between-group differences on any REM sleep parameters. For instance, some previous PTSD studies have reported altered REM percentage (e.g., Lavie et al., 1979; Engdahl et al., 2000; Lipinska et al., 2014) and increased REM density (e.g., Dow et al., 1996; Raboni et al., 2014). One way to account for the discrepancy between our findings and others regarding REM sleep in PTSD relates to variability in the chronicity of the condition. Mellman et al. (2014) demonstrated that REM-associated symptoms change from early diagnosis to chronic PTSD (i.e., when the diagnosis has been present for a relatively short time, REM sleep tends to be shorter and more fragmented, with longer latency to enter the stage, but when the diagnosis has been present for several years, REM sleep tends to be longer, with shorter latency to enter the stage). In the current study, there was relatively large variation in the number of years participants had carried the diagnosis (range = 6–61 months, *SD* = 12.00). This variability might have acted to decrease the effects detected by between-group analyses of, for instance, group means related to REM latency.

Further toward replicating the circumstances under which sleep-state misperception might be proposed, we analyzed data regarding everyday (past month) subjective sleep quality, as measured by the PSQI. Our analyses suggested that PTSD-diagnosed individuals reported more disruptions than TE and HC individuals.

Taken together, the PSG (objective, laboratory) data and the PSQI (subjective, home) data suggest that there might be sleep-state misperception in PTSD-diagnosed individuals: that they self-report experiencing poor sleep quality, but objectively sleep relatively normally. No such difference between objective and subjective sleep quality was demonstrated for TE and HC participants.

Our further analyses provide an alternative perspective, however. Analyses of Laboratory PSQI data detected no between-group differences in subjective sleep quality in the laboratory. A follow-up analysis suggested that this lack of between-group differences could be attributed to the fact that, within the PTSD group only, self-reported sleep quality in the laboratory was significantly better than that at home. Furthermore, with the exception of one individual, PTSD-diagnosed participants scored well above the PSQI cut-off score indicating clinical sleep disruption (range = 3–19, where the cut-off is 5). However, the range of Laboratory PSQI scores was quite different (0–12), with 12 PTSD-diagnosed participants falling at or below the clinical cut-off.

These results suggest that, on average, PTSD-diagnosed individuals slept better in the laboratory than in their home environment, whereas participants in the TE and HC groups experienced similar sleep quality across the two environments. Participant comfort in the laboratory is borne out by the fact that the vast majority (52/60) indicated that the electrodes and other PSG equipment did not influence their sleep quality at all.

Despite the lack of routine measurement of subjective sleep quality comparing the two environments, several studies have noted that PTSD-diagnosed individuals may sleep better in the laboratory than at home because they may perceive the former environment as safer (Hurwitz et al., 1998; Spoormaker and Montgomery, 2008; Kobayashi et al., 2012). The authors of those studies speculate that a perception of safety may lead to decreased overall anxiety and hyperarousal, and therefore increased sleep quality. In the present study, participants' qualitative responses regarding differences in their sleep quality across the two environments suggested that many attributed their better sleep quality in the laboratory to a perception that that environment was safer, quieter, more comfortable, and freer from distractions than their home environment.

This interpretation is supported by data from Herbst et al. (2010), who compared two nights of PSG recording in the laboratory to two nights of identical recording at home. They found that when PTSD-diagnosed participants, in comparison with healthy controls, experienced their first recording night at home, they had higher REM density, which is a sign of REM dysregulation. However, this effect was not observed when PTSD-diagnosed participants spent their first night in the sleep laboratory. These results suggest that the home environment, rather than the laboratory environment, may be associated with a greater degree of sleep disruption in PTSD.

The participants in this study all lived in low-SES communities. Many lived in shanty towns, in over-crowded houses, with constant all-night activity on the roads outside. They often co-slept with other family members. Nighttime criminal activities, such as house break-ins, are common in their communities. Therefore, the quiet single-occupant environment of the sleep laboratory differed substantially from their home sleep environments, and it is quite plausible that sleep quality would be rated as better in the former than in the latter.

Finally, a series of correlational analyses allowed us to further debunk the notion of sleep-state misperception in the current sample. Those analyses suggested that, although everyday subjective sleep quality (as measured by the PSQI) was not significantly correlated with objective sleep measures (as measured by PSG), there was significant congruence between laboratory subjective sleep quality (as measured by the Laboratory PSQI) and objective sleep measures. This pattern of associations was especially strong for PTSD-diagnosed participants. For example, in those individuals, poor laboratory subjective sleep quality was associated with less sleep efficiency, more time spent awake after sleep onset, and more frequent awakenings.

Hence, although we did observe a disjuncture between one set of subjective measures and the objective measures, we also observed consistency between the other set of subjective measures and the objective measures. This, then, is not evidence for sleep-state misperception.

Our conclusions are tempered by the following limitations. First, we measured home sleep quality using the PSQI, which evaluates sleep quality over a month-long period, whereas we measured laboratory sleep quality using the Laboratory PSQI and PSG outcomes, taken over a single night. This discrepancy in the time-scale across which measures in the different environments were taken is a methodological confound. Our study does not, therefore, rule out the possibility that PTSD-diagnosed individuals might experience marked sleep disruption over the period of a month, but might not experience any major disruption on any single night (Cox et al., 2017).

Second, we did not take objective measures of sleep in the home environment. The inclusion of such measures would have allowed us to evaluate, directly, the effects of environmental context on sleep quality in PTSD-diagnosed individuals. Ideally, of course, one would use identical polysomnographic measures in the laboratory, and the home, but this is complicated by technological limitations (e.g., placement and quantity of electrodes, and method of electrode placement, is relatively restricted in home-based EEG applications). Furthermore, polysomnographic recording is particularly challenging when participants are drawn from communities where their, and the researchers', safety at night cannot be guaranteed. Finally, the introduction of a device, a particular new set of behaviors, and the knowledge of external monitoring into the home sleep environment may not guarantee a valid representation of everyday sleep.

Future studies examining sleep quality in PTSD-diagnosed individuals should be cognizant of how (objective vs. subjective measurement), where (in the home vs. laboratory environment) and for how long (a single vs. multiple nights of measurement) sleep quality is measured. Furthermore, researchers should record sleep quality in such a way as to be able to comment on whether polysomnographic recordings are representative of everyday sleep quality. In addition to polysomnographic measurement in the laboratory, recording subjective sleep quality in both the home and the laboratory environment can be a useful, convenient method of understanding the relationship between everyday sleep and sleep under laboratory conditions. For example, if laboratory objective, laboratory subjective, and everyday subjective measures of sleep all correlate strongly with each other, then objective measures are more likely to represent everyday sleep accurately. Controlling for the environment and duration of measurement is critical to avoid attributing the discrepancy between subjective and objective measures of sleep quality in PTSD to individual characteristics rather than to methodological confounds.

## Summary and conclusion

Numerous previous studies have highlighted inconsistencies between subjective and objective reports of sleep disruption in PTSD-diagnosed individuals, demonstrating that these individuals typically self-report experiencing poor sleep quality, but objectively appear to experience comparatively minimal sleep disruption. To account for these inconsistencies, some authors hypothesize that PTSD-diagnosed individuals have sleep-state misperception; that is, they perceive their sleep to be more disrupted than it objectively is. Here, we demonstrated that differences in environmental context, and not sleep-state misperception, is the likeliest explanation for the discrepancy between objective and subjective measures of sleep quality in PTSD. Specifically, we found that PTSD-diagnosed individuals felt safer in the laboratory than in their home environments, where violence and crime are an everyday reality, and that therefore they experienced fewer sleep disruptions during a night of laboratory sleep than they reported experiencing during sleep at home. We conclude that studies of sleep in PTSD must critically evaluate and account for methodological confounds that might affect results, rather than attributing inconsistent findings to individual patient characteristics.

## Author contributions

GL, Conceptualized the project, collected and analyzed the data and prepared the manuscript for publication. KT, Provided support for conceptualization and prepared the manuscript for publication.

### Conflict of interest statement

The authors declare that the research was conducted in the absence of any commercial or financial relationships that could be construed as a potential conflict of interest.
